# Vitamin D is a prognostic marker in heart failure

**Published:** 2010-12

A Dutch study of vitamin D levels in patients with heart failure, presented at the 2010 European Society of Cardiology (ESC) congress in Stockholm, provoked considerable interest among cardiologists as it suggests that they advise their patients to maintain appropriate vitamin D levels.^1^

Liu and co-workers at the University Medical Centre, Groningen, Netherlands showed that:

- a low vitamin D concentration is associated with a poor prognosis in heart failure
- the correlation found between vitamin D, plasma renin activity and C-reactive protein suggests that an activated renin–angiotensin system and an altered cytokine profile may play an important role in the association between vitamin D and heart failure.

In this study, 548 patients hospitalised due to heart failure were studied, and the correlation was examined between 25-hydroxy vitamin D (nmol/l) levels, plasma renin activity (PRA), the presence of inflammatory cytokines, and the incidence of death or heart failure re-hospitalisations during the 18-month follow up.

The mean age was 71 years and mean left ventricular ejection fraction was 33 ± 14%. Patients were grouped into tertiles according to vitamin D levels (T1: < 29.6; T2: 29.6–43.9; T3: > 43.9 nmol/l). Vitamin D levels were higher in males (p < 0.001) and younger patients (p = 0.002), and those with lower galectin-3 (p < 0.001) and NT-proBNP levels (p < 0.001).

Multivariate linear regression analysis showed that plasma renin activity (p = 0.003) and C-reactive protein (CRP) levels (p = 0.002) are independent predictors of vitamin D levels. During follow up, 165 patients died and 142 were re-hospitalised. Kaplan-Meier analysis showed that lower vitamin D concentration was associated with an increased risk for all-cause mortality (log rank test, p = 0.014) and increased risk for the combined endpoint (mortality and heart failure re-hospitalisation; log rank test, p = 0.045).

Importantly, after adjustment in a multivariable Cox regression analysis, high vitamin D concentration remained independently associated with a decreased risk for all-cause mortality (HR: 0.66; 95% CI: 0.44–0.99; p = 0.044) and the combined endpoint (HR: 0.69; 95% CI: 0.51–0.95; p = 0.022).

The results of this study provide compelling evidence that a high vitamin D status is associated with improved survival in heart failure patients. ‘It seems that physicians should advise their heart failure patients to maintain their vitamin D levels by taking supplements, eating oily fish or eggs or ensuring exposure to sunlight’, Ms Liu conluded.
